# Ontogenic Changes of Villus Growth, Lactase Activity, and Intestinal Glucose Transporters in Preterm and Term Born Calves with or without Prolonged Colostrum Feeding

**DOI:** 10.1371/journal.pone.0128154

**Published:** 2015-05-26

**Authors:** Julia Steinhoff-Wagner, Ulrike Schönhusen, Rudolf Zitnan, Monika Hudakova, Helga Pfannkuche, Harald M. Hammon

**Affiliations:** 1 Institute of Nutritional Physiology “Oskar Kellner”, Leibniz Institute for Farm Animal Biology (FBN), 18196 Dummerstorf, Germany; 2 Institute of Nutrition, National Centre of Agriculture and Food Nitra, 04181 Kosice, Slovakia; 3 School of Economics and Management in Public Administration, 85104 Bratislava, Slovakia; 4 Institute of Veterinary-Physiology, Leipzig University, 04103 Leipzig, Germany; Oregon State University, UNITED STATES

## Abstract

Oral glucose supply is important for neonatal calves to stabilize postnatal plasma glucose concentration. The objective of this study was to investigate ontogenic development of small intestinal growth, lactase activity, and glucose transporter in calves (n = 7 per group) that were born either preterm (PT; delivered by section 9 d before term) or at term (T; spontaneous vaginal delivery) or spontaneously born and fed colostrum for 4 days (TC). Tissue samples from duodenum and proximal, mid, and distal jejunum were taken to measure villus size and crypt depth, protein concentration of mucosa and brush border membrane vesicles (BBMV), total DNA and RNA concentration of mucosa, mRNA expression and activity of lactase, and mRNA expression of sodium-dependent glucose co-transporter-1 (SGLT1) and facilitative glucose transporter 2 (GLUT2) in mucosal tissue. Additionally, protein expression of SGLT1 in BBMV and GLUT2 in crude mucosal membranes and immunochemical localization of GLUT2 in the enterocytes were determined. Villus height in distal jejunum was lower in TC than in T. Crypt depth in all segments was largest and the villus height/crypt depth ratio in jejunum was smallest in TC calves. Concentration of RNA was highest in duodenal mucosa of TC calves, but neither lactase mRNA and activity nor SGLT1 and GLUT2 mRNA and protein expression differed among groups. Localization of GLUT2 in the apical membrane was greater, whereas in the basolateral membrane was lower in TC than in T and PT calves. Our study indicates maturation processes after birth for mucosal growth and trafficking of GLUT2 from the basolateral to the apical membrane. Minor differences of mucosal growth, lactase activity, and intestinal glucose transporters were seen between PT and T calves, pointing at the importance of postnatal maturation and feeding for mucosal growth and GLUT2 trafficking.

## Introduction

With birth nutrient supply changes from parenteral energy supply by the placenta to oral supply with milk feeding. The gastrointestinal tract (GIT) develops rapidly in the weeks before birth and maturation of GIT is induced by the prenatal glucocorticoid surge [1–3]. Cortisol promotes growth and maturation of organs around birth and increases small intestinal weight as well [4], whereas administration of cortisol to ovine fetus during late gestation affects cell proliferation, but fails to influence enterocyte morphology [5]. After birth, growth and development of the GIT is affected by feeding, especially by colostrum intake [6–8].

Energy supply switches during the perinatal period from glucose and amino acids (fetal) to colostrum and milk with fat as main energy source. However the supply of glucose (lactose from milk) is important for neonates to stabilize postnatal blood glucose concentrations [9,10] and to ensure the supply of tissues that depend on glucose metabolism [11]. Lactose is the main nutritive carbohydrate source in neonatal mammals, which is split into glucose and galactose by the intestinal enzyme lactase. Lactase is present in the bovine fetus and its activities increase from day 90 to 180 of gestation [12]. Intake of colostrum may stimulate small intestinal lactase activity in calves after birth, as this is the case in other species [13,14]. The intestinal absorption of glucose occurs predominantly in the duodenum and jejunum [15] by active transport at the apical side whereby sodium-dependent glucose co-transporter 1 (SGLT1) transports monosaccharides from the intestinal lumen across the brush border membrane in a competitive process [15]. Glucose and galactose transport continues across the basolateral membrane by facilitated glucose transport (GLUT2) [15]. Glucose transport can be influenced by feeding [16] and it has been assert that the intrinsic activity of GLUT2 increases with elevated luminal glucose concentration and by trafficking of GLUT2 to the apical membrane [17–19], although this concept is still under discussion [20].

Preterm born calves, rather than mature born calves, suffer from hypoglycemia and the prenatal glucose metabolism is impaired, indicated by reduced endogenous glucose production [21] and a reduced response to colostrum feeding [8,22,23]. Although glucose transporters seems to be less influenced by cortisol concentrations after birth [24], the inadequate cortisol surge in preterm born calves may be one cause for insufficient intestinal development at birth and impaired postnatal maturation and functioning [8]. Based on these premises, we tested the hypothesis that calves delivered preterm have impaired villus growth as well as reduced lactase activity and intestinal glucose transporter expression when compared to full-term born calves and that postnatal maturation and colostrum intake stimulates lactase activity and glucose transporters in the small intestine of neonatal calves. Therefore, preterm calves were delivered 9 d before term by caesarean section to avoid exposure to final cortisol surge at end of gestation [4]. Villus growth, lactase activity, and intestinal glucose transporters were investigated 2 h after milk intake when postprandial digestion and absorption of glucose is activated due to feed intake. The aim of the present study was to identify differences in postprandial glucose digestion and absorption in calves with variable gestation length and stage of postnatal maturation.

## Materials and Methods

### Animals and feeding

The experimental procedures were carried out according to the animal care guidelines and were approved by the relevant authorities of the State Mecklenburg-Vorpommern, Germany (LALLF M-V/TSD/7221.3–1.1-014/07).

German Holstein calves were studied. Calves (n = 7 per group) were either born preterm (PT), or at term (T) and were slaughtered 26 h after birth, or born at term, fed colostrum for 4 days, and were slaughtered on day 4 of life (TC). Term born calves (T and TC; all male) were spontaneously born at nearby commercial farms (Landwirtschaftlicher Milchhof “Am Recknitztal” eG, Alte Dorfstraße 5, 18299 Laage, Germany, and Landwirtschaftsgesellschaft Prisannewitz mbH, Am Bahnhof 8a, 18196 Scharstorf, Germany) by vaginal delivery and were transported immediately after birth to the experimental facility. Preterm calves (PT; one female, six males) were delivered by caesarean section 9 days before expected parturition at the operation room of the institute. All calves were born as singletons from multiparous cows. Calves were kept in single boxes at the research station of the University of Rostock (Dummerstorf, Germany) and housed at 19°C room temperature with straw bedding and free access to water.

Calves of PT and T did not receive milk during first day of life. Calves of group TC received previously collected pooled colostrum obtained from milkings 1, 3, and 5 (days 1, 2, and 3 after parturition, respectively) on their first 3 days of life. Composition of the three colostrum milks were published previously [25]. Fed amounts (per meal) of colostrum in group TC were 4% of body weight (BW) on day 1, and 5% of BW from day 2 on. Calves of group TC were fed by bottle twice daily (8.00 h and 16.00 h) if not otherwise indicated. All calves were fed colostrum at 5% of BW of colostrum (milking 5) 2 h before slaughtering, i.e. 26 h after birth for calves of group PT and T and on day 4 for calves of group TC. To ensure that all calves received an equal amount of colostrum, calves with reduced appetite were tube fed their targeted portion.

Colostrum was collected before the study started. Cows housed at the farm where calves were born were milked twice daily and the colostrum of milkings 1, 3, and 5 after parturition was stored separately in plastic bottles at −20°C. Individual colostrums pools from milkings 1, 3, and 5 were prepared at the beginning of the study and then stored in plastic bottles at −20°C until used to provide the same milk. Before feeding, colostrum was warmed in a water bath at 40°C. All calves were injected with antibiotics subcutaneously (25 mg Enrofloxacin per 10 kg BW; Baytril 5%; Bayer AG, Leverkusen, Germany) on day 1 and TC additionally on days 2 and 3. To avoid iron-deficiency, calves received intramuscular injections of iron dextran (10 mg per kg BW; Ursoferran 100, Serumwerk Bernburg AG, Bernburg, Germany). The health status was evaluated daily based on the following clinical traits: rectal temperature, heart rate, respiratory rate, behavior, nasal discharge, respiratory sounds, fecal consistency, and navel inspection. Navels were disinfected with Betadine (Mundipharma Medical Company, Hamilton, Bermuda) after birth to protect calves from infection. Body weight was determined immediately after birth and before slaughter [21].

### Tissue sampling

Calves were slaughtered by stunning with a captive bolt pistol and exsanguinating 2 h after feeding at 26 h after birth (PT, T) or on day 4 of life (TC). Small intestine was removed and lumen was cleaned with ice-cold physiological saline solution. Tissue samples were taken from mid duodenum and proximal, mid and distal jejunum, frozen in liquid nitrogen, and stored at -80°C until analyzed. Additionally, samples of described small intestine segments were opened longitudinally and mucosa samples were scrapped off with a microscope slide, snap-frozen in liquid nitrogen, and stored at -80°C until analyzed. Additional tissue samples from respective segments were opened as pieces of 1 cm^2^ and fixed in 4% formaldehyde solution for morphometric measurements or in Histofix (Roth, Karlsruhe, Germany) for immunohistochemistry.

### Morphometric Measurements

Formaldehyde fixed samples were rinsed in water, dehydrated in a graded series of ethanol (30%, 50%, 70%, and 90%), cleared in benzene, and saturated with and embedded in paraffin. Sections of 5 μm thickness (10 slices of each sample) were stained with haematoxylin/eosin and investigated under light microscope (Axiolab, Carl Zeiss Jena, Germany). The height, circumference, and cut surface area of 30 villi and depth of 30 crypts were determined by the computer-operated *Image C* picture analysis system (Imtronic GmbH, Berlin, Germany) and the IMES analysis program by using a color video camera (SONY 3 CCD, Sony Electronics Ltd., Tokyo, Japan) [26].

### Lactase Activity Assay

The activity of lactase (EC 3.2.1.23.) was measured according to Mir et al. [27]. Briefly, frozen mucosal tissue (200 mg sample homogenized in 1 ml saline for 3 min) was washed into a test tube using two 2.5 ml aliquots of saline solution (0.9% NaCl, wt/vol). Only 100 ml of the homogenate was transferred to a reaction tube and placed in a water bath (37°C), then 400 μL of 56 mM lactose in citrate buffer (pH 6.6, 0.01 M) was added, respectively. After shaking and incubation for 30 min, enzyme activity was stopped in boiling water. The tubes were centrifuged (2000 × *g*, 15 min at 4°C). Galactose produced during the incubation with tissue homogenate was measured to determine the activity of lactase. Concentration of galactose in the supernatant was determined by the UV method (UV Test Lactose/D-Galactose No. 1763030, Boehringer Mannhein/R-Biopharm, Mannheim, Germany). Enzyme activity was expressed as μM substrate hydrolyzed per 30 min at 37°C.

### Quantification of SGLT1 and GLUT2 mRNA by Real-Time PC

Frozen mucosa samples were ground in liquid nitrogen immediately prior to analysis. Total DNA and RNA were extracted using TRIzol Reagent (Invitrogen, Karlsruhe, Germany), re-suspended in RNase-free water, and quantified by UV absorption. The integrity and purity of RNA were tested by measurement of optical density (ratio at 260 and 280 nm being greater than 1.9) and by electrophoresis using ethidium bromide staining. A total of 1 μg RNA was reverse transcribed into cDNA using random primers (Random Primers, Invitrogen, Karlsruhe, Germany) and purified using High Pure PCR Product Purification Kit (Roche Diagnostics, Mannheim, Germany) [28].

Real time RT-PCR was performed by LightCycler (Roche Molecular Biochemicals, Mannheim, Germany) using SYBR Green I as fluorescence dye [28]. Specific primers were used to measure the mRNA concentrations of SGLT1, GLUT2, lactase, ribosomal protein S9 (RPS9), and β-actin. Sequences of all primers are published [28–30]. Primers were designed to flank a region that contains at least one intron to ensure that no contaminating genomic DNA was amplified that could lead to false signals.

Melting temperatures that were performed by the LightCycler in a melting curve analysis program after the last amplification cycle demonstrated specific PCR products. Upon agarose gel electrophoresis, all PCR products moved in one single band and showed the expected size [28–30]. Products for sglt1 and β-actin were verified by sequencing using an ABI Sequencing kit (ABI Big Dye Terminator, Applied Biosystems, Darmstadt, Germany) and an ABI 310 Genetic Analyzer (Applied Biosystems, Darmstadt, Germany).

Quantification of mRNA was performed by relative expression using mean of β-actin and RPS9 as reference gene transcript [28,31]. Efficiencies of PCR were > 1.8 and expression of the reference gene was not affected by ontogenic development or intestinal segment. Inter- and intraassay coefficient of variation for RT-PCR of target and reference genes were below 1%, respectively.

### Measurements of SGLT1 and GLUT2 protein

Preparation of brush-border membrane vesicles (BBMV) were performed as described [32] with few modifications [29]. The final protein concentration in BBMV was 10–20 mg/ml. Portions (200 μL) of BBMV were frozen in liquid nitrogen and stored until analyzed. Purity of BBMV was determined by marker enzymes: alkaline phosphatase activity (ALP, EC3.1.3.1; kit no. 816388 Roche Diagnostics, Mannheim, Germany) and ouabain-sensitive Na+-K+-activated ATPase (Na+-K+-ATPase, EC 3.6.1.3) [33] were determined. Because on protein basis ALP was 23-fold and Na+-K+-ATPase was 1.4-fold more enriched in BBMV suspensions than in the corresponding homogenates, concentrations of BBM in final BBMV preparations were satisfactory [32]. Protein concentrations of BBMV were determined according to the Bradford [34] method with BSA (Sigma-Aldrich Co., St. Louis, US) as standard. For the preparation of crude cell membranes mucosa tissue was homogenized and centrifuged as described [29].

Protein expression of SGLT1 in BBMV as well as GLUT2 in cell crude membranes were quantified by SDS PAGE (7.5% acrylamide) and immunoblot analyses [29]. Gel electrophoresis was performed using a Mini-PROTEAN Electrophoresis System (BIO-RAD Laboratories GmbH, München Germany) and protein markers (Precision Plus Protein, BIO-RAD Laboratories GmbH) as molecular mass standard. Proteins were transferred from the gel onto nitrocellulose membrane (GE Healthcare, Buckinghamshire, UK) and blocked with TBS-T (20 mM Tris-HCl, pH 7.6, 137 mM NaCl, and 50 ml/L Tween 20) containing 0.5% non fat dry milk powder (Carl Roth GmbH, Karlsruhe, Germany). Membranes were incubated overnight at 4°C with rabbit anti SGLT1 (1:1,000, BIOTREND, Köln, Germany) or with rabbit anti GLUT2 (1:750, BIOTREND), respectively. After washing membranes with TBS-T (3 ×15 min), they were incubated 1 h at room temperature with horseradish peroxidase-conjugated sheep anti-rabbit IgG (1:10,000, Serotec, Kidlington, Oxford, UK) followed by visualizing with chemiluminescence (ECL Western Blotting Analysis System, GE Healthcare). The blot was autoradiographed (Hyperfilm ECL, Amersham Bioscience, Buckinghamshire, UK) and quantified by scanning the densitometry (Molecular Imaging Systems version 4.0, Eastman Kodak Company, Rochester, NY). Data were normalized to control preparations and reported as relative arbitrary units.

### Immunohistochemistry

Specimen fixed in Histofix were washed (3 × 10 min) with 0.1 M phosphate buffered saline (PBS) 24 h after sampling. Immunohistochemical labeling was performed on Cryostat sections after incubation overnight at 4°C in PBS containing 30% sucrose. Transversal sections (15 μm) were cut using a cryostat, mounted on poly-L-lysine coated slides, and stored at -20°C. Sections were processed for fluorescence immunohistochemistry by washes (3 × 10 min) in PBS and pre-incubation for 60 min in buffer solution (PBS containing 4% horse serum and 0.5% Triton X-100) [35]. This buffer solution was also used for dilution of primary and secondary antibodies. After pre-incubation, the tissues were incubated with rabbit-anti-GLUT2 (sc-9117, Santa Cruz, Heidelberg, Germany) primary antibodies in a dilution of 1:50 for 18 h at room temperature. The tissues were then washed in PBS (3 × 10 min) before they were incubated in buffer solution containing the secondary antibodies. Secondary anti-rabbit IgG raised in donkeys and conjugated to Cy2 (Dianova, Hamburg, Germany) were used in a dilution of 1:200. In some additional stainings, specifity of the binding between primary and secondary antibodies was proven by omitting the primary antibodies. After the final washings the specimens were coverslipped with a solution of NaHCO_3_/Na_2_CO_3_ (0.5 M, pH 7.0) containing 0.1% NaN_3_ and 80% glycerol. The stained sections were analyzed using an epifluorescence microscope (IX50, Olympus, Tokyo Japan) with a black and white video camera (F-view, Olympus, Hamburg, Germany) attached to an image analysis system (cell F, Olympus, Hamburg, Germany). From each animal and intestinal compartment stained two sections were analyzed. Three photomicrographs were taken from each section. The magnification used was 200-fold and the exposure time was 200 ms. Immunoreactivity was evaluated separately for apical and basolateral membranes of the epithelial cells in an arbitrary scale from 0 (no immunoreaction) to 3 (highest immunoreaction) in intervals of 0.5.

### Statistics

The statistical analysis was done with the program SAS (version 9.3; SAS Institute Inc., Cary, NC) using the procedure Mixed Model with ontogenic group (PT, T, and C) and intestinal segment as fixed effects and the individual calf as random effect. For all tests differences with P values < 0.1 were defined as trend and with P values < 0.05 were assessed to be significant. Ranks representing histological immunoreactivity were compared separately for each gut segment in regard to different ontogenic groups with Wilcoxon Two-Sample Test using the SAS program.

## Results

### Mucosal growth

Histomorphometrical results were shown in Table 1.Villus circumference and area were almost exclusively lowest in duodenum of all groups when compared to other gut segments. There was a trend for greater overall villus circumference in T than in TC. Villus height of PT and T was greater in mid jejunum than in duodenum, and for TC was greater in mid jejunum than in distal jejunum. Besides an overall group effect, villus height in distal jejunum was higher in T than in TC. Crypt depth decreased along the small intestine in PT and T, but not in TC, and was greatest in TC when compared to PT and T. The ratio of villus height to crypt depth increased along the small intestine in PT and T, but not in TC, and was lowest in jejunum of TC when compared to PT and T.

**Table 1:** Histomorphometric measurements in small intestinal mucosa of calves.

	Groups			ANOVA (P-values)		
Item^1^ ^,^ ^2^	PT	T	TC	Group	Segment	Group × Segment
Villus circumference, *μm*						
Duodenum	1404 ± 35 ^b^	1455 ± 32 ^b^	1384 ± 24 ^b^	0.06	0.0001	0.9
Proximal Jejunum	1631 ± 46 ^a^	1645 ± 36 ^a^	1553 ± 36 ^a^			
Mid Jejunum	1679 ± 53 ^a^	1706 ± 28 ^a^	1594 ± 39 ^a^			
Distal Jejunum	1631 ± 31 ^a^	1660 ± 25 ^a^	1594 ± 24 ^a^			
Villus area, *μm* ^*2*^						
Duodenum	68697 ± 3454 ^b^	69877 ± 2083 ^b^	66647 ± 1765 ^b^	0.15	0.0001	1
Proximal Jejunum	79863 ± 2907 ^a^	80278 ± 2187 ^a^	76552 ± 2407 ^a^			
Mid Jejunum	82545 ± 3154 ^a^	84704 ± 3505 ^a^	79710 ± 2293 ^a^			
Distal Jejunum	79173 ± 2298 ^a^	81796 ± 1801 ^a^	74023 ± 1242 ^a,b^			
Villus height, *μm*						
Duodenum	600 ± 20 ^b^	617 ± 10 ^b^	575 ± 14 ^a,b^	0.01	0.0001	0.4
Proximal Jejunum	665 ± 23 ^a,b^	671 ± 19 ^a,b^	593 ± 18 ^a,b^			
Mid Jejunum	692 ± 32 ^a^	701 ± 29 ^a^	654 ± 23 ^a^			
Distal Jejunum	650 ± 21 ^a,b,A,B^	672 ± 17 ^a,b,A^	561 ± 13 ^b,B^			
Crypt depth, *μm*						
Duodenum	181 ± 8 ^a,B^	197 ± 5 ^a,B^	226 ± 7 ^a,A^	0.0001	0.0001	0.0001
Proximal Jejunum	148 ± 3 ^b,B^	160 ± 4 ^b,B^	230 ± 7 ^a,A^			
Mid Jejunum	125 ± 5 ^b,c,B^	137 ± 6 ^b,c,B^	196 ± 4 ^b,A^			
Distal Jejunum	112 ± 3 ^c,B^	126 ± 5 ^c,B^	235 ± 8 ^a,A^			
Villus height/crypt depth						
Duodenum	3.4 ± 0.2 ^c^	3.2 ± 0.1 ^c^	2.6 ± 0.1	0.0001	0.0001	0.0001
Proximal Jejunum	4.5 ± 0.2 ^b,A^	4.2 ± 0.1 ^b,A^	2.6 ± 0.1 ^B^			
Mid Jejunum	5.6 ± 0.4 ^a,A^	5.2 ± 0.4 ^a,A^	3.4 ± 0.2 ^B^			
Distal Jejunum	5.8 ± 0.3 ^a,A^	5.4 ± 0.3 ^a,A^	2.4 ± 0.1 ^B^			

^1^Histomorphometric measurements were performed in duodenum, proximal, mid, and distal jejunum 2 h after feeding on day 2 of life in preterm (PT) and term (T) born calves and on day 4 of life in term born and colostrum-fed calves (TC).

^2^Values are means ± standard error, n = 7 per group, different values between groups (within a row) are shown with capital letters (^A,B^ *P* < 0.05) and between segments (within one column) of each parameter are shown with lowercase letters (^a,b,c^ *P* < 0.05).

Mucosal protein concentration decreased along the small intestine in PT and T, but the decrease was less clear and not significant in TC (Table 2). The overall trend for a group effect of mucosal protein pointed at slightly greater concentrations in PT calves. Vesicle protein showed a significant group × segment interaction with group-specific variation of vesicle protein along the small intestine. Concentration of DNA did not show any differences with respect to group and gut segment. The overall RNA concentrations indicated significant group and segments effects and in duodenum RNA concentrations were greater in TC than in PT and T.

**Table 2:** Protein, DNA, and RNA content in small intestinal mucosa of calves.

	Groups			ANOVA (P-values)		
Item^1^ ^,^ ^2^	PT	T	TC	Group	Segment	Group × Segment
Mucosal Protein, *mg/g mucosa*						
Duodenum	87.5 ± 6.8 ^a^	74.1 ± 2.7 ^a^	63.8 ± 6.2	0.1	0.0001	0.12
Proximal Jejunum	73.3 ± 5.0 ^a,b^	67.7 ± 3.6 ^a,b^	60 ± 4.3			
Mid Jejunum	70.3 ± 4.9 ^a,b^	69.1 ± 3.6 ^a,b^	69.3 ± 9.6			
Distal Jejunum	65.2 ± 3.9 ^b^	52.2 ± 1.9 ^b^	56.5 ± 3			
Vesicle Protein, *mg/g tissue*						
Duodenum	0.79 ± 0.22 ^a,b^	0.71 ± 0.2	0.44 ± 0.05 ^b^	0.17	0.001	0.04
Proximal Jejunum	1.02 ± 0.1 ^a^	0.85 ± 0.1	0.75 ± 0.05 ^a,b^			
Mid Jejunum	0.78 ± 0.09 ^a,b^	0.55 ± 0.07	0.97 ± 0.1 ^a^			
Distal Jejunum	0.5 ± 0.04 ^b^	0.57 ± 0.03	0.36 ± 0.06 ^b^			
DNA, *μg/mg mucosa*						
Duodenum	5.0 ± 0.8	3.6 ± 0.5	3.9 ± 0.4	0.3	0.2	0.7
Proximal Jejunum	3.2 ± 0.2	2.9 ± 0.6	3.7 ± 0.4			
Mid Jejunum	4.2 ± 1.0	3.0 ± 0.2	3.7 ± 0.2			
Distal Jejunum	3.3 ± 0.6	3.2 ± 0.3	3.9 ± 1.0			
RNA, *μg/mg mucosa*						
Duodenum	1.5 ± 0.2 ^B^	1.8 ± 0.2 ^B^	4.8 ± 0.5 ^A^	0.0001	0.04	0.1
Proximal Jejunum	3.5 ± 0.5	3.3 ± 0.3	4.8 ± 0.3			
Mid Jejunum	2.8 ± 0.3	3.4 ± 0.3	4.1 ± 0.6			
Distal Jejunum	3 ± 0.2	3.2 ± 0.5	4.1 ± 0.3			

^1^Measurements were performed in duodenum, proximal, mid, and distal jejunum 2 h after feeding on day 2 of life in preterm (PT) and term (T) born calves and on day 4 of life in term born and colostrum-fed calves (TC).

^2^Values are means ± standard error, n = 7 per group, different values between groups (within a row) are shown with capital letters (^A,B^ *P* < 0.05) and between segments (within one column) of each parameter are shown with lowercase letters (^a,b^ *P* < 0.05).

### Lactase gene expression and activity

Lactase mRNA expression of all groups was lowest in distal jejunum (Table 3). Lactase activity expressed per mucosal mass was lowest in distal jejunum of all groups and was greater in proximal jejunum than in mid jejunum of PT and T, but not TC. Lactase activity expressed per mucosal mass in distal jejunum tended to be greater in TC than in PT. Lactase activity expressed per mucosal protein concentration in PT and TC was lowest in distal jejunum and in T was greatest in proximal jejunum.

**Table 3:** Gene expression and activity of lactase in small intestinal mucosa of calves.

	Groups			ANOVA (P-values)		
Item^1^ ^,^ ^2^	PT	T	TC	Group	Segment	Group × Segment
Lactase mRNA related to reference gene						
Duodenum	3.85 ± 2.09	7.67 ± 3.1	7.45 ± 2.98	0.2	0.0001	0.6
Proximal Jejunum	4.54 ± 1.58	6.34 ± 1.35	8.24 ± 3.06			
Mid Jejunum	2.5 ± 1.35	2.62 ± 0.42	8.63 ± 3.44			
Distal Jejunum	0.09 ± 0.03	0.62 ± 0.19	2.79 ± 1.55			
Lactase Activity, *μmol/(30 min mg mucosa)*						
Duodenum	484 ± 56 ^a,b^	411 ± 62 ^b^	386 ± 49 ^a,b^	0.2	0.0001	0.001
Proximal Jejunum	565 ± 55 ^a^	665 ± 71 ^a^	475 ± 51 ^a^			
Mid Jejunum	305 ± 24^b^	447 ± 39 ^b^	460 ± 40 ^a^			
Distal Jejunum	20 ± 4 ^c^	184 ± 28 ^c^	230 ± 50 ^b^			
Lactase Activity *μmol/(30 min mg mucosal protein)*						
Duodenum	5.84 ± 0.95 ^a^	5.6 ± 0.81 ^b^	6.2 ± 0.82 ^a,b^	0.12	0.0001	0.07
Proximal Jejunum	7.92 ± 0.93 ^a^	10.06 ± 1.19 ^a^	8.46 ± 1.37 ^a^			
Mid Jejunum	4.55 ± 0.6 ^a^	6.51 ± 0.55 ^b^	7.99 ± 1.82 ^a^			
Distal Jejunum	0.3 ± 0.05 ^b^	3.54 ± 0.56 ^b^	4.13 ± 0.97 ^b^			

^1^Measurements were performed in duodenum, proximal, mid, and distal jejunum 2 h after feeding on day 2 of life in preterm (PT) and term (T) born calves and on d 4ay of life in term born and colostrum-fed calves (TC).

^2^Values are means ± standard error, n = 7 per group, different values between segments (within one column) of each parameter are shown with lowercase letters (^a,b,c^ *P* < 0.05).

### Glucose Transporters in the Small Intestine

Expression of SGLT1 mRNA and SGLT1 protein in apical vesicles decreased along the small intestine, but indicated no significant group differences (Table 4). Expression of GLUT2 mRNA showed lowest abundance in distal jejunum and in PT, GLUT2 mRNA was higher in proximal jejunum than in distal jejunum. Protein expression of GLUT2 in total membrane indicated lowest concentration in mid jejunum.

**Table 4:** Gene and protein expression of glucose transporters in small intestinal mucosa of calves.

	Groups			ANOVA (P-values)		
Item^1^ ^,^ ^2^	PT	T	TC	Group	Segment	Group × Segment
SGLT1 mRNA related to reference gene						
Duodenum	9.5 ± 5	16 ± 5.4	36.6 ± 14.9	0.15	0.01	0.9
Proximal Jejunum	17.9 ± 11	18.6 ± 8.6	37 ± 12.6			
Mid Jejunum	10 ± 4.1	8.6 ± 2.7	23.7 ± 8.8			
Distal Jejunum	5.5 ± 2.4	3.7 ± 1.1	19.4 ± 10.6			
SGLT1 in Apical Vesicles, *μmol/(30 min mg mucosa)*						
Duodenum	3.34 ± 1.61	1.49 ± 0.47	2.94 ± 0.56	0.17	0.01	0.3
Proximal Jejunum	3 ± 0.66	1.24 ± 0.32	1.11 ± 0.31			
Mid Jejunum	1.53 ± 0.49	1.61 ± 0.31	1.28 ± 0.15			
Distal Jejunum	1.05 ± 0.16	1.05 ± 0.28	0.99 ± 0.14			
GLUT2 mRNA related to reference gene						
Duodenum	1.85 ± 1.1 ^a,b^	1.13 ± 0.31	1.43 ± 0.8	0.5	0.01	0.1
Proximal Jejunum	5.31 ± 2.75 ^a^	1.3 ± 0.25	1.5 ± 0.67			
Mid Jejunum	2.68 ± 1.77 ^a,b^	1.11 ± 0.3	1.65 ± 0.7			
Distal Jejunum	0.39 ± 0.13 ^b^	0.25 ± 0.11	0.29 ± 0.14			
GLUT2 in Total Membrane, *OD*						
Duodenum	1.1 ± 0.2	1.12 ± 0.13	1.06 ± 0.12	0.7	0.01	0.6
Proximal Jejunum	1.1 ± 0.07	1.04 ± 0.12	0.85 ± 0.16			
Mid Jejunum	0.63 ± 0.18	0.64 ± 0.28	0.49 ± 0.19			
Distal Jejunum	1.06 ± 0.26	0.57 ± 0.18	0.93 ± 0.26			

^1^ Gene and protein expression of glucose transporters SGLT1 and GLUT2 were performed in duodenum, proximal, mid, and distal jejunum 2 h after feeding on day 2 of life in preterm (PT) and term (T) born calves and on day 4 of life in term born and colostrum-fed calves (TC).

^2^Values are means ± standard error, n = 7 per group, different values between segments (within one column) of each parameter are shown with lowercase letters (^a,b^ *P* < 0.05).

**Fig 1:**
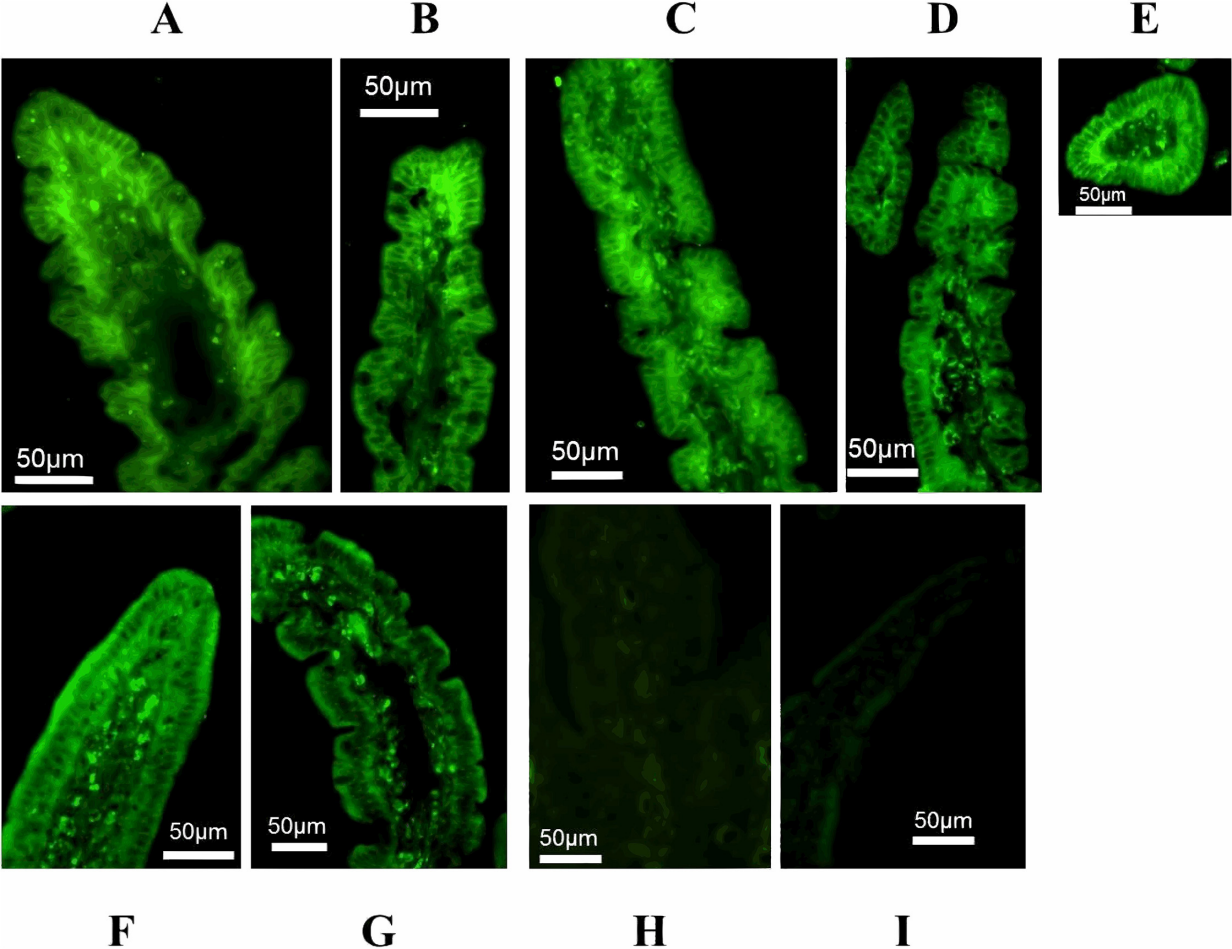
Immunofluorescence analysis of GLUT2 protein in small intestinal mucosa of calves. Protein expression of GLUT2 is shown in duodenum (A) and proximal jejunum (B) of preterm born calves (PT), in proximal jejunum (C), mid jejunum (D), and duodenum (E) of term born calves (T), and in proximal (F) and mid jejunum (G) of 4-day old and colostrum-fed calves (TC). Immunofluorescence analysis was performed without anti-GLUT2 antibody in duodenum (H; PT calf) and mid jejunum (I; TC calf).

Immunofluorescence labeling of GLUT2 in duodenum, proximal, and mid jejunum of PT and T were greater basolateral than apical, and in proximal jejunum of TC were lower basolateral than apical. Furthermore, GLUT2 labeling indicated that number of GLUT2 transporters were greater in the apical membrane, but lower in the basolateral membrane in TC than in T and PT (Fig 1, Table 5).

**Table 5:** Rank of immunoreactions of GLUT2 in small intestinal mucosa of calves.

	Groups			Kruskal Wallis Test (P-values)		
Item^1^ ^,^ ^2^	PT	T	TC	PT vs. T	PT vs. TC	T vs. TC
GLUT2 Fluorescence, apical						
Duodenum	0.69 ± 0.18	0.78 ± 0.19	0.61 ± 0.18	ns	ns	ns
Proximal Jejunum	0.57 ± 0.19	0.53 ± 0.25	1.36 ± 0.18	ns	0.01	0.05
Mid Jejunum	0.43 ± 0.19	0.8 ± 0.19	1.64 ± 0.16	ns	0.01	0.05
Distal Jejunum	0.21 ± 0.16	0.21 ± 0.19	0.86 ± 0.16	ns	0.01	0.01
GLUT2 Fluorescence, basolateral						
Duodenum	2.32 ± 0.13*	2.15 ± 0.14*	0.42 ± 0.13	ns	0.001	0.001
Proximal Jejunum	2.6 ± 0.14*	2.83 ± 0.18*	0.82 ± 0.13*	ns	0.001	0.001
Mid Jejunum	2.37 ± 0.14*	2.43 ± 0.14*	1.48 ± 0.12	ns	0.001	0.001
Distal Jejunum	0.12 ± 0.12	0.26 ± 0.14	0.44 ± 0.12	ns	0.001	0.001

^1^Rank of immunoreactions of GLUT2 in apical and basolateral membranes were performed in duodenum, proximal, mid, and distal jejunum 2 h after feeding on d 2 of life in preterm (PT) and term (T) born calves and on d 4 of life in term born and colostrum-fed calves (TC).

^2^Values are means ± standard error, differences between two groups (within a segment, ns = non-significant) or between location (apical versus basolateral, * *P* < 0.05) are tested with Kruskal Wallis Test.

## Discussion

Villus growth in our study changed with postnatal maturation, mainly by postnatal development and colostrum feeding. Previous findings indicated stimulation of villus growth by nutritive and non-nutritive components of colostrum in neonatal calves [36–38]. Interestingly, crypt depth increased, but villus size and the villus height/crypt depth ratio decreased in the small intestine during postnatal development in term born calves [23], which supports present findings. Colostrum feeding for 4 days likely caused enhanced abrasion, crypt fission, enhanced migration rate of crypt epithelial cells to villus tips, reduced apoptosis, and increased survival rate [8]. These implications of colostrum feeding might explain changes in villus growth and the higher RNA concentrations in the mucosa of the small intestine in 4-day old calves. In addition, there is a negative association of villus size and crypt depth, where intestinal cell proliferation takes place. This negative association illustrates negative feedback control of intestinal epithelial growth [39,40]. In our study the reduced villus height/crypt depth ratio in TC calves indicated enhanced functioning of the small intestinal mucosa in 4 day old calves when compared to 2-d old preterm and full-term born calves [8].

Differences in villus growth and crypt depth between PT and T calves were minor, which partly contrasts previous findings in preterm and full-term born calves [23]. However, PT calves in our study were slaughtered one day after birth and two hours after feeding colostrum, whereas PT and T calves in the study of Bittrich et al. [23] were slaughtered directly after birth without food intake. Feeding, especially colostrum feeding, may have affected mucosal growth and proliferation in PT calves, although feeding responses on postnatal mucosal growth and maturation of the small intestine were reduced in PT calves when compared to full-term born calves [23]. Nonetheless, our hypothesis that in calves delivered by section 9 days before term with a reduced exposure to the prenatal glucocorticoid surge villus size and crypt depth is impaired when compared to full-term born calves was not valid. Previous findings in preterm pigs and calves, where exposure to glucocorticoid treatment supports maturation of the gastrointestinal tract, indicated the importance of the perinatal glucocorticoid status on villus growth and development [1,3,4]. We can not exclude that in our preterm born calves the reduced late fetal exposure to glucocorticoids was insufficient to impede villus growth.

In contrast to villus growth, lactase gene expression and enzyme activity in small intestinal enterocytes did not differ between 2-day and 4-day old calves. Although lactase gene expression is numerically lowest in enterocytes of PT calves the capacity for lactose digestion might not be affected by an impaired lactase synthesis in PT calves, and changes in villus size might be more important for determination of digestive capacity in neonatal calves than effects on intestinal enzymes [23,38]. Elevated lactase activity is already present around birth [12,23]. The same is true for glucose transporter gene expression and protein density. Both transporters are present in enterocytes at birth, but neither SGLT1 nor GLUT2 gene and protein expression differed in the small intestinal mucosa of 2-day and 4-day full-term born calves. This was in contrast to findings in piglets, where GLUT2 protein abundance was higher in 2-day old than in newborn piglets [24]. One reason for the absence of differences of SGLT1 and GLUT2 in small intestinal enterocytes during the neonatal period might be that all calves were fed colostrum with same amounts of lactose 2 h before slaughtering. According to SGLT1 protein it is well known that protein expression is regulated by the amount of available glucose [15,16].

Interestingly, GLUT2 localization in small intestinal enterocytes markedly changed with ongoing postnatal development. Two hours after feed intake, more GLUT2 was localized in the apical than in the basolateral membrane of 4-day old calves and GLUT2 protein at the basolateral membrane was lower, but on the apical membrane was greater in 4-day than in 2-day old calves, either born full-term or preterm. It is important to note that feed intake, and especially lactose intake, 2 h before tissue sampling was the same among groups. Obviously, postprandial GLUT2 trafficking from the basolateral to the apical membrane increased with postnatal maturation of the small intestine. Although still under discussion, it is assumed that GLUT2 trafficking from the basolateral to the apical membrane support intestinal glucose absorption in polarized epithelial cells and this GLUT2 trafficking is stimulated by elevated luminal glucose concentrations [18,19]. Therefore, we suppose that the increased GLUT2 trafficking in 4-day old calves contributes to postnatal maturation of the small intestine. This maturation effect might be age-dependent and stimulated by colostrum feeding. However, recent investigations on postprandial GLUT2 localization in small intestinal enterocytes of calves revealed no feeding effect on apical GLUT2 protein localization when a milk-based formula was fed instead of colostrum for 4 days of life [38].

Postprandial glucose plasma concentrations were greater in 4-day than 2-day old full-term born calves, which may support the idea that intestinal capacity for glucose absorption was greater in 4-day than in 2-day old calves [21]. It is well known that due to intestinal maturation by feeding, postnatal glucose absorption increases with time [6]. Due to experimental studies on endogenous glucose production in these calves, T and PT calves were not fed immediately after birth [38]. We cannot rule out that the lack of colostrum intake at birth in PT and T calves may have led to impaired intestinal maturation with less trafficking of GLUT2 to the apical membrane, although GLUT2 and SGLT1 protein concentrations in small intestine were not affected by this feeding protocol. In any case, term born calves fed no colostrum on first day of life had reduced plasma concentration of glucose during first week of life, although they received colostrum from the second day of life on [41].

On the other hand, basolateral and apical localization of GLUT2 protein did not differ between PT and T calves. In addition, in both groups GLUT2 protein localization was greater in the basolateral than in the apical membrane. Obviously, the maturation processes at the end of gestation and first milk intake did not provide a sufficient signal to trigger GLUT2 trafficking to the apical membrane in neonatal calves, although we have found some GLUT2 protein in the apical membrane of PT and T calves. Alternatively, more than one meal is needed to stimulate GLUT2 trafficking into the apical membrane. There were also no differences with respect to SGLT1 and GLUT2 gene and protein expression in PT and T calves throughout the small intestine and, therefore, no difference in intestinal glucose absorption was expected between both groups. Surprisingly, postprandial changes of plasma glucose concentration indicated an increase 2 h after feeding in PT, but not in T calves [21]. Provided that most of the glucose comes from milk lactose and with respect to the minor endogenous glucose production in PT when compared to T calves [21], glucose absorption in PT calves was obviously not impaired and basic functioning of lactose ingestion seems to be established in preterm born calves. Although not significantly different, lactase activity per mucosal protein was numerically reduced in PT calves when compared to T calves. It was previously shown that lactase activity in preterm pigs can be stimulated by cortisol infusion and feed intake, whereas feeding has no effect in term born piglets [1,3]. This might indicate the importance of the perinatal glucocorticoid status and feeding on intestinal glucose absorption in preterm born mammals.

However, it remains to be clarified why in term born calves there was no such postprandial increase of plasma glucose [21]. We speculate that the first-pass glucose uptake in the splanchnic tissue might be different between preterm and term born calves and that an increase in plasma glucose was essential for preterm calves, because these calves showed marked hypoglycemia 24 h after birth and a reduced endogenous glucose production when compared to mature calves [21]. An additional explanation might be that glucose utilization in small intestinal enterocytes is different between PT and T calves. Differences in mucosal glucose utilization during the neonatal period were described for piglets [24].

Changes of lactase activities and glucose transporters along the small intestine indicated greatest densities in the proximal part of the small intestine, which fits well to common literature [6,12,42,43]. With regard to morphometric changes along the small intestine it is notable that changes of the villus height/crypt depth ratio were seen in PT and T calves, but not in TC calves. These findings illustrate that differences in postnatal maturation of the intestine depend on gut localization and impaired maturation may mainly occur in the distal parts of the intestine in 2-day old calves, either born preterm or at term. Probably, the maturation process is related to feed intake and the response on feed intake in the distal parts increases when calves are fed for several days.

In conclusion, our study clearly demonstrates that maturation processes after birth mainly affects morphometric parameters and the trafficking of GLUT2 from the basolateral to the apical membrane in small intestinal enterocytes. On the contrary, fewer differences in mucosal growth and development of small intestinal mucosa were seen when comparing preterm born and term born calves one day after birth. Therefore, early postnatal maturation and feeding have a great impact on small intestinal functioning and may support glucose absorption in calves. Enhanced postprandial GLUT2 trafficking in intestinal mucosa may contribute to the elevated glucose status in TC calves [21].
